# End-to-End Implicit Object Pose Estimation

**DOI:** 10.3390/s24175721

**Published:** 2024-09-03

**Authors:** Chen Cao, Baocheng Yu, Wenxia Xu, Guojun Chen, Yuming Ai

**Affiliations:** School of Computer Science and Engineering, Wuhan Institute of Technology, Wuhan 430073, China

**Keywords:** deep learning for visual perception, pose estimation, implicit representation

## Abstract

To accurately estimate the 6D pose of objects, most methods employ a two-stage algorithm. While such two-stage algorithms achieve high accuracy, they are often slow. Additionally, many approaches utilize encoding–decoding to obtain the 6D pose, with many employing bilinear sampling for decoding. However, bilinear sampling tends to sacrifice the accuracy of precise features. In our research, we propose a novel solution that utilizes implicit representation as a bridge between discrete feature maps and continuous feature maps. We represent the feature map as a coordinate field, where each coordinate pair corresponds to a feature value. These feature values are then used to estimate feature maps of arbitrary scales, replacing upsampling for decoding. We apply the proposed implicit module to a bidirectional fusion feature pyramid network. Based on this implicit module, we propose three network branches: a class estimation branch, a bounding box estimation branch, and the final pose estimation branch. For this pose estimation branch, we propose a miniature dual-stream network, which estimates object surface features and complements the relationship between 2D and 3D. We represent the rotation component using the SVD (Singular Value Decomposition) representation method, resulting in a more accurate object pose. We achieved satisfactory experimental results on the widely used 6D pose estimation benchmark dataset Linemod. This innovative approach provides a more convenient solution for 6D object pose estimation.

## 1. Introduction

Object pose estimation plays a crucial role in various fields such as augmented reality, robotic grasping, and autonomous driving [1].In these domains, there is an increasing demand for high-precision and real-time perception of object position and orientation [2,3]. Moreover, object pose estimation is pivotal in many applications, including virtual reality gaming [4], human pose estimation [5], and robotic grasping, thus garnering increasing attention in the field of computer science.

With the advancement in deep learning, traditional pose estimation methods, such as treating pose estimation as a classification task or using template matching, are gradually being replaced by deep learning-based approaches due to their efficiency and high accuracy [6]. Currently, most researchers address the 6D pose estimation task in two stages: first, extracting latent feature information from the images and estimating dense 2D-3D correspondences, and then solving the corresponding pose information through PnP (Perspective-n-Point)/RANSAC (Random Sample Consensus), followed by refining the pose information using ICP (Iterative Closest Point) [7,8]. Although two-stage algorithms can achieve high accuracy, they also pose several challenges. For instance, two-stage algorithms are non-differentiable, making it difficult to directly use end-to-end networks. Furthermore, these algorithms are computationally intensive and often fail to perform well in practical applications. Additionally, when processing images with machines, the images are discretized into pixels, and the intermediate feature maps are treated similarly. When using convolution, upsampling is required to match the feature maps of different scales, which can blur the precise information learned within the feature maps [9].

To address these issues, we draw inspiration from the field of implicit representation and propose a feature map implicit representation method suitable for pose estimation. We integrate this method with bidirectional feature fusion pyramids [10] and use implicit representation to estimate the feature maps, forming our proposed highly scalable module, the Implicit Feature Pyramid Network (IFPN). This module encodes the coordinate information of the feature map using implicit feature expression and establishes a connection with the original feature information to infer the feature information of feature maps at different scales. Finally, we introduce three branches for different tasks, namely regressing the object bounding box, object category, and 6D pose. In the 6D pose regression, we leverage our proposed uncertain IFPN module to regress the implicit information onto the object’s 2D-3D mapping and the probability of the object surface area. We believe that early fusion of the two types of features will lose the local information of each feature and interfere with both at the global level. Therefore, we propose a dual-stream network to separately extract the features of the two types of information. We use bidirectional lateral connections to map them into a 9D space and then constrain the uncertain information through pose information constraints, providing greater tolerance and better fitting performance.

In conclusion, while two-stage algorithms remain predominant in the field of pose estimation, they exhibit several shortcomings. The non-differentiability of decoupling R and T regression hinders the direct regression from source data to the final result, making end-to-end regression methods more compelling. Neural network training is challenged by variations in feature map sizes, impacting the accuracy of feature maps. Lastly, the non-differentiability of object rotation is addressed by directly regressing the rotation matrix through a 2D-3D vector field, resulting in more accurate rotation outcomes. Our contributions include addressing these challenges.

1. We extend implicit neural representation to pose estimation, introducing a reusable and scalable feature map implicit expression module: IFPN. The uncertainty of this module provides a more stringent constraint for subsequent pose estimation networks, leading to improved pose estimation results.

2. We combine the proposed feature map implicit expression module with the feature pyramid and introduce a novel end-to-end pose estimation network. This network exhibits strong scalability and achieves favorable results.

3. For regressing the 6D pose of objects, considering the correlation and independence between different input information, we propose a bidirectional fusion-based DP-PnP method to address the information loss and interference caused by the coupling between different information sources.

## 2. Related Work

### 2.1. Two-Stage Algorithm

Traditional algorithms employ two-stage approaches, initially estimating the correspondence between 2D and 3D, followed by the application of RANSAC/PnP algorithms to determine object poses [7,8,11,12,13] Most commonly, they first employ semantic segmentation models to isolate specific objects, subsequently combining other information to solve for 6D poses. For instance, PVNet [6] divides the network regression task into two stages: keypoint extraction and uncertainty-aware PnP. Pix2pose [14] predicts the 3D coordinates of each object from RGB images, estimates the relative error between 3D coordinates using an encoder, pixel-wise predicts the 2D-3D correspondence, and then directly predicts the object’s 6D pose using PnP. Additionally, it restores occluded parts through generative adversarial networks, rendering robustness to occluded object pose estimation. BB8 [15] and YOLO-6D [16] initially fix 2D image keypoints, project the vertices of 3D bounding boxes onto the 2D plane, and then employ PnP to solve for object 6D poses. These methods utilize dense correspondences which offer better accuracy compared to sparse correspondences. While exhibiting favorable performance, such methods are characterized by slower processing speeds.

### 2.2. End-to-End Algorithms

Two-stage algorithms currently demonstrate superior performance; however, such methods are not amenable to differentiable tasks. Conversely, end-to-end direct regression methods offer the advantage of speed but lack accuracy [17]. SSD-6D [18] extends target detection from the 2D plane to 3D, estimating depth values from bounding box sizes and then inferring the object’s 6D pose from the highest-scoring 2D bounding box, inferred viewpoints, and in-plane rotations from the network. Xiang et al. [7] first calculate offsets from annotated image keypoints to centroid points, then estimate centroid points using Hough voting while simultaneously estimating depth values from 2D images to establish 2D-3D correspondences, and finally regress to the object’s 6D pose directly. Additionally, researchers have introduced differentiable PnP solutions for such problems, e.g., differentiable PnP [17,19,20], making the second stage of two-stage algorithms differentiable, enabling the end-to-end training of pose. Consequently, the PnP module becomes part of the neural network, facilitating end-to-end pose estimation via learnable 2D-3D correspondences. Although faster, such methods exhibit lower accuracy. Training with larger model networks theoretically yields better results, but strict hardware requirements and the need for both accuracy and speed in this research domain pose constraints. To enhance the accuracy of end-to-end methods, researchers consider the inherent inaccuracies in directly estimating rotation components. Zhou et al. [21] propose representing rotations in dimensions higher than 5D, ensuring continuity in rotation representation, thereby replacing traditional representations like axis–angle and quaternions, consequently enhancing the network’s regression capability. Furthermore, J et al. [22] discuss the feasibility of upgrading 3D rotation SVD orthogonalization within networks, suggesting projecting the rotation group onto SVD for training, yielding more advanced performance.

### 2.3. Implicit Neural Representation

Implicit neural representations based on neural networks exploit the fact that neural networks are universal function approximators. Coordinate-based MLPs (multilayer perceptrons) are widely employed to represent continuous-domain signals in computer vision and graphics, and such methods have been shown to be more accurate than traditional approaches [23,24,25,26] Moreover, utilizing such methods can reduce data requirements, as these MLPs provide an efficient framework for data storage, with the memory footprint of storing MLPs being independent of the resolution of the feature maps. In the recent field of 3D reconstruction, object shapes and scenes can be represented using MLPs, encoding coordinates and mapping them to signals, thereby achieving Implicit Neural Representations (INRs) [27,28,29,30]. DeepSDF [27] represents shapes by learning distance functions, while Nerf [29] synthesizes novel views of complex scenes through a novel approach, both making significant contributions to 3D reconstruction. Ref. mgh [30] maps the latent space of digits to 2D shapes, replacing the periodic activation functions within the MLPs of implicit neural representations with Relu to improve modeling quality. LiiF [31] utilizes implicit neural expressions to model continuous images. We embed implicit neural representations into our network and fuse them with feature pyramid networks to smoothly integrate feature maps of different scales.

## 3. Method

### 3.1. Overview

**Figure 1:**
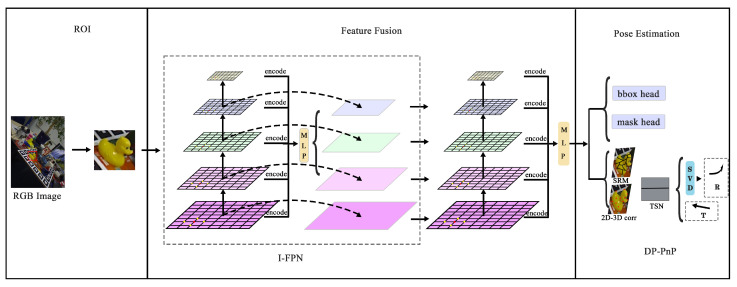
Our network architecture comprises three main components. Initially, we utilize pre-trained detectors to extract ROIs (regions of interest). These ROIs are then fed into the feature pyramid for feature fusion. This stage includes the I-FPN (Implicit-Feature Pyramid Networks) and regression modules. I-FPN encodes feature maps of various scales and constructs continuous feature maps using implicit expression functions. The regression module inputs this implicit information into a multilayer perceptron (MLP) to estimate feature maps of different scales. Subsequently, the fused implicit information is used to directly estimate the required pose information via the MLP, including bbox (bounding boxes) and masks, which are employed to estimate the 2D bounding box and pixel categories, respectively. This information aids in predicting the pose information, specifically rotation *R* and translation *T*. Additionally, we regress the implicit information into object surface information SRM and the mapping information between 2D and 3D. Through the designed two-stream network TSN fusion, the rotation information represented by 9DSVD and the translation information is output.

We embed the implicit representation of feature maps into the feature pyramid network to address the issue of inaccurate feature extraction at different scales. As shown in Figure 1, our framework first extracts features at various scales. Due to the varying scales of different feature maps, traditional methods use upsampling for decoding. Instead, we map the feature maps into a function using implicit representation, thereby avoiding the information loss and inaccuracies caused by upsampling. Additionally, we utilize MLPs to obtain the implicit representation of feature maps at different scales. This uncertainty can better constrain the pose regression information. Finally, we extract the implicit representation of feature maps at different scales again. We then regress the implicit information to the object’s bounding box, classification, and surface probability, and the mapping between 2D and 3D. We employ a dual-stream network to fuse the surface probability and the intermediate features during the regression process of 2D-3D mapping. This fusion of the two streams means that they complement each other, resulting in a more robust and accurate outcome.

### 3.2. Implicit Representation of Feature Maps

Implicit Features: The most significant challenge in aggregating information from multi-scale feature maps is the fusion of features with different resolutions. Typically, upsampling of feature maps is required to align them to the same resolution. By employing implicit representation of feature maps, we can construct a continuous feature map without the need for upsampling [32,33,34,35]. Inspired by recent advances in implicit neural representations in 3D reconstruction and image super-resolution, we leverage an implicit feature function to build a continuous feature map. Implicit feature functions are commonly modeled using MLPs to fit a decoding function for obtaining the continuous feature map M. Given a discrete feature map, where feature vectors correspond to pixels in the image, each feature vector is considered uniformly distributed in the two-dimensional space, assigning a two-dimensional coordinate to each feature. The representation of each code in the feature map is defined as in Equation (1).

(1) $$M\left(x_{q}\right)=f_{\theta }\left(p^{1},p^{2},\ldots ,p^{n},x_{q}-x^{1},x_{q}-x^{2},\ldots ,x_{q}-x^{n}\right)$$

where $p^{1},p^{2},\ldots ,p^{n}$ represent the latent codes of the *n* nearest feature points, and $x_{1},x_{2},\ldots ,x_{n}$ denote the coordinates of these latent codes, respectively. We construct a decoding function $f_{\theta }$ learned in conjunction with the feature extractor to precisely represent the continuous feature field.

Position Encode: Implicit neural representations have a limitation in that standalone MLPs are more adept at learning low-frequency components and struggle to capture high-frequency information, a phenomenon known as the positional bias. Our proposed solution is to encode the input information by projecting input coordinates into a high-dimensional Fourier feature space. The positional encoding function we use is given by Formula (2).

(2) $$\varphi \left(x\right)=(\sin\left(\omega_{1}x\right),\cos\left(\omega_{1}x\right),\sin\left(\omega_{2}x\right),\cos\left(\omega_{2}x\right),\ldots ,\sin\left(\omega_{d/2}x\right),\cos\left(\omega_{d/2}x\right))$$

where the frequency *d* is initialized to $\omega_{d/2}=2e^{d/2}$ and the final positional encoding function is given by Formula (3).

(3) $$M\left(x_{q}\right)=f_{\theta }\left(p^{n},\varphi \left(x_{q}-x^{n}\right),x_{q}-x^{n}\right)$$

Feature Alignment: Inspired by the continuous representation of images, one approach to feature alignment is to transform each layer’s feature map into a continuous feature map, allowing direct access to the feature values at any point on the feature map for coordinate alignment. To simplify this process, we fuse all the required feature maps to model, thus simplifying the acquisition of continuous feature maps for each scale.

We combine the continuous implicit feature maps into an overall feature map model, directly defining the continuous feature map *M* from discrete feature maps at different resolutions, where i represents the number of feature layers. Specifically, the value of *M* in $x_{q}$ is given by Formula (4).

(4) $$M\left(x_{q}\right)=f_{\theta }\left({\left\{p_{i}^{n}\right\}}_{i=1}^{5},{\left\{\varphi \left(x_{q}-x_{i}^{n}\right),x_{q}-x_{i}^{n}\right\}}_{i=1}^{5}\right)$$

## 4. Implicit Feature Fusion Network

**Figure 2:**
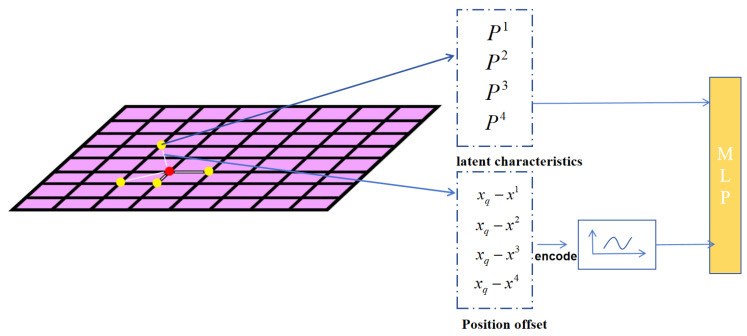
In the I-FPN module, the red points represent the desired feature points, and the yellow points represent the nearest four feature points around the points to be estimated. The offset information is encoded and then, along with the actual feature values, input into the MLP, forming our I-FPN module.

Implicit Feature Network: We take the differently scaled feature maps obtained from the encoder as input and output feature maps at arbitrary scales. Traditional encoders require upsampling to match the features, but we believe upsampling can lead to information loss. Therefore, we treat upsampling as a learned process, employing an MLP to solve a function, denoted as *f*. Taking the encoder input $x(x_{1},x_{2},x_{3},x_{4},x_{5})$ as different-scale feature maps from the encoder, we use *x* as input and obtain $f_{x}$ as the estimated feature map at different scales. Since MLPs struggle to leverage deep dimensional features, we encode the input information and feed it into the MLP to estimate feature maps at different scales. We then fuse the estimated feature maps with the original feature maps from the feature pyramid to ensure the presence of original data. Finally, we obtain fused feature maps at different scales. This process is encapsulated in a module called IFPN, which can be repeated *n* times to thoroughly integrate different-scale feature information. Refer to Figure 2 for an illustration of this process.

As shown in Figure 2, we take the four nearest points around the feature point to be estimated. The original feature values of these four points, along with their offset information, are encoded. Subsequently, all values are fused and input into the MLP, thus constructing an implicit model. This model establishes a mapping relationship, allowing us to obtain the feature information of the point to be estimated based on its coordinate information.

Branch Networks: Applied to input feature maps of various scales into an MLP. To streamline operations, unlike the IFPN, the MLP regression outputs are directly fed into various branch networks. Notably, in the semantic segmentation branch, the MLP regression results are directly mapped to classification values for each pixel. For the pose regression network, we introduce an RT regression branch aimed at mapping implicit information obtained from feature maps to the correlation between 2D and 3D spaces. Inspired by GDR- Net [18], we regress the implicit information to the low-level dense graph first, establishing a connection between the low-level dense graph and the 2D pixel coordinates to obtain the mapping information between 2D and 3D. Additionally, we regress the implicit information to the probability distribution of object surface areas, thereby obtaining a coarse pose estimation and making judgments regarding the symmetry of the object. Furthermore, our designed DP-PnP utilizes the mapping information to obtain the 6D pose information.

### 4.1. DP-PnP

**Figure 3:**
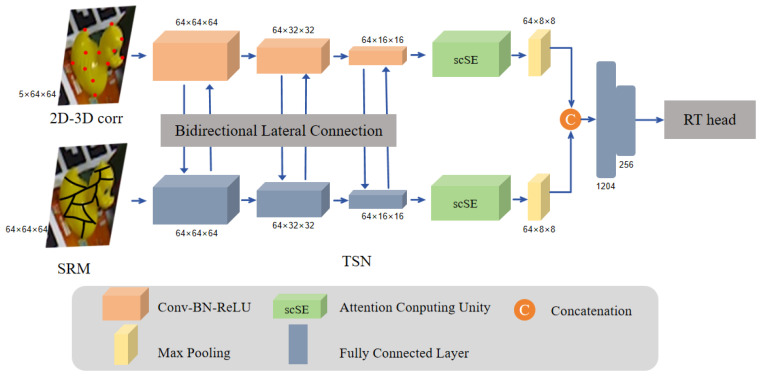
TSN is a dual-encoder network where two encoders interact horizontally to regress object poses through self-attention layer connections. SRM (Spatial Relationship Model) represents the classification of pixels in each region. The black lines delineate different region classifications within the yellow duck. The red dots on the image correspond to key points on the 3D object mapped to points on the 2D object. Both types of information are processed through the same encoder, horizontally interconnected, and fused via self-attention layers to yield the pose estimation results through fully connected layers.

GDR proposed a novel approach to achieve end-to-end regression of object 6D poses using differentiable PnP. Traditionally, PnP methods are unable to directly regress 6D poses. Their method involves connecting the estimated object surface information with the 2D-3D mapping and passing it as input to the proposed Patch-PnP, enabling differentiable PnP. We argue that simply connecting the object surface information and the 2D-3D mapping as input would lead to loss of independently held information, thereby impacting the estimation results. To address this issue, we introduced a bidirectional fusion network to facilitate interaction between the two streams. Instead of directly merging the two features, we fused the features from earlier stages, enhancing the inter-stream connectivity. Additionally, we incorporated an attention mechanism into the bidirectional network using concurrent scSE [36] modules. The scSE modules can extract crucial spatial and channel information from input feature maps and can efficiently introduce attention into the model with minimal additional parameters. The specific network architecture is depicted in Figure 3. In this architecture, the traditional surface probability distribution and 2D-3D mapping are separately inputted into two small convolutional neural networks. Initially, a 1 × 1 convolution is used to output blocks with the same channels. Then, the two feature maps are horizontally concatenated element-wise. Subsequently, the feature maps are reduced by half in both spatial dimensions and channel numbers using 3 × 3 convolutions with a stride of 2. Similarly, this block undergoes bidirectional connections. After passing through the scSE attention modules, the feature maps are further reduced by half using a 2 × 2 max pooling layer with a stride of 2. Finally, the concatenated bidirectional features are fed into a fully connected layer, serving as the input to the RT block.

Regarding R, we project it onto the SVD of the rotation group. Analysis suggests that simply replacing the commonly used representation with the SVD orthogonalization process can lead to more advanced performance [22]. Based on this theory, we represent rotation as a 3 × 3 matrix, namely a 9D representation. For *T*, we directly regress to a 3D representation, thereby obtaining the object’s 6D pose.

(5) $$SVD\left(M\right)=U\Sigma V^{T}$$

### 4.2. The Loss Function and Pose Estimation

We have proposed five loss functions, namely $L_{b},L_{c},L_{i},L_{k},L_{p}$. The final loss function is formulated as follows:

(6) $$L=\lambda_{1}L_{b}+\lambda_{2}L_{c}+\lambda_{3}L_{i}+\lambda_{4}L_{k}+\lambda_{5}L_{p}$$

where $\lambda_{1},\lambda_{2},\lambda_{3},\lambda_{4},\lambda_{5}$ correspond to the weight of the respective loss function.

Here, $L_{b}$ is the bounding box loss, utilizing IOU loss to constrain the generation of the two-dimensional object bounding box. $L_{c}$ is the class loss, which is also the semantic segmentation loss, primarily referring to [33]. $L_{i}$ is the loss employed to constrain the implicit module, as defined in Equation (7).

(7) $$L_{i}=\frac{1}{n}\sum_{i}^{n}\left|p_{i}-f_{\theta }\left(i_{x},i_{y}\right)\right|$$

We employ L1 loss to constrain the feature maps generated by the implicit module, where the points p(1) are not generated by upsampling but are pixels estimated through implicit representation. These are the coordinates of the coordinate map, obtained from the estimated mapping by the MLP. Using this constraint can enhance the stability and accuracy of the model.

$L_{k}$ stands for Lucas–Kanade. It is a method used to estimate the motion of a set of 2D points in an image sequence. The keypoint positions in the image are predicted, with the 3D points obtained through CAD rendering, thus establishing the 2D-3D mapping information. The specific formula for this process is as follows, where $kp_{ij}$ is the ground-truth 2D keypoint location, n is the number of 3D keypoints, m is the number of 2D correspondences of $kp_{i}$, and *M* is the number of total 2D correspondences predicted by our network in the image.

(8) $$L_{k}=\frac{1}{M}\sum_{i=1}^{n}\sum_{j=1}^{m}∥kp_{ij}-kp_{i}^{*}∥$$

$L_{p}$ is our final 6D pose prediction function, inspired by PoseCNN. The specific formula is given by Equation (9).

(9) $$L_{p}=\frac{1}{n}\sum_{i=1}^{n}∥\left(R^{*}p_{i}+t^{*}\right)-\left(Rp_{i}+t\right)∥$$

where $R^{*}$ and $t^{*}$ are the estimated rotation matrix and translation vector, and R and t are the ground-truth ones.

## 5. Experiment

In this section, we validate the effectiveness of our proposed modules and branch networks through experiments. We test our method on common public 6D pose datasets. We compare our approach with end-to-end pose estimation methods and two-stage estimation methods in terms of estimation performance and speed. We design several ablation experiments to verify the effectiveness of each component proposed in our method.

### 5.1. Dataset

The Linemod dataset serves as a standard benchmark for object 6D pose estimation, encompassing 13 low-texture objects across 13 videos, along with annotated 6D poses and instance masks. This dataset annotates the 6D pose of a single object, despite the presence of other objects in the scene. To demonstrate the superior performance of our method, we have also devised a multi-object pose detection approach.

For multi-object detection, we utilize the Occluded Linemod dataset, a subset of the Linemod dataset. This dataset annotates the 6D poses of multiple objects within single-object scenes, with many instances featuring occlusions. This complexity renders pose estimation challenging, thereby showcasing the efficacy of our method.

### 5.2. Metrics

We use the common evaluation metric ADD to assess the effectiveness of our method. ADD measures the average distance between the 3D model points transformed by the predicted pose and the 3D model points obtained through the ground-truth pose. When the distance is less than 10% of the model’s diameter, it indicates that the estimated pose is correct. We follow and evaluate symmetric objects using the ADD(-S) metric, which measures the deviation from the nearest model point. The predicted pose is represented as $\left[R^{*},t^{*}\right]$, and the ground-truth pose is represented as [R,t], where *x* is one of the *m* vertices on the model. When not considering the symmetry of the object, our evaluation metric is given by Formula (10).

(10) $$ADD=\frac{1}{m}\sum_{x\epsilon O}∥\left(R_{x}+t\right)-\left(R_{x}^{*}+t^{*}\right)∥$$

where *m* is the number of points on the object point cloud, *O* is the object point cloud data, and the pairs *R* and *t* represent the true rotation and translation components as well as the estimated rotation and translation components. When considering the symmetry of the object, our evaluation metric for the target is defined as Formula (11), where $x_{1}$ and $x_{2}$ correspond to the estimated points closest to the true points, respectively.

(11) $$ADD-S=\frac{1}{m}\sum_{x_{1}\epsilon O}\min_{x_{1}\epsilon O}∥\left(R_{x}+t\right)-\left(R_{x}^{*}+t^{*}\right)∥$$

### 5.3. Implementation Details

In our experiments, we implemented it using TensorFlow and trained it for a total of 500 epochs. The learning rate was set to 0.1. We had 5 hyperparameters in the experiment. We found that the best results were achieved when they were set to $\lambda_{1}=\lambda_{2}=1,\lambda_{3}=\lambda_{4}=\lambda_{5}=0.05$. We believe that the classification loss and bounding box loss are easier to estimate and should account for a larger proportion. In addition, during the experiment, we configured implicit learning of relative positions to find the four points closest to the considered feature point, which improved the estimation performance. In addition, we used a method denoted as ϕ to control the model depth and width, setting them to 0 and 3, respectively. We refer to the method of setting hyperparameters of efficientdet, but we only modify the backbone network depth and the number of layers of I-FPN to compare the superiority of our method. In the process of 6D pose regression, in order to improve network efficiency and simplicity, we directly regress implicit information into our network branch.

#### 5.3.1. Evaluation on the Linemod Dataset

**Figure 4:**
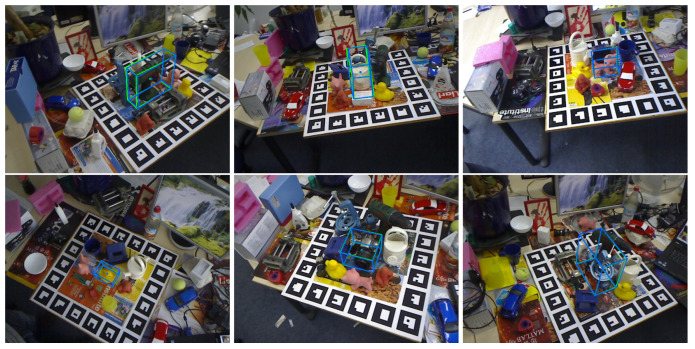
This is the experimental result figure for detecting the 6D pose of a single object, where the blue represents the estimated results and the green represents the ground truth.

On the Linemod dataset, we primarily focus on detecting individual objects. Figure 4 illustrates the six selected objects for detection. Table 1 presents the results of our experiments, comparing our method with major models based on the Linemod dataset. As shown in Table 1, our approach based on implicit learning significantly improves the estimation accuracy. Although the accuracy for some objects is lower compared to RNNPose, our method performs well on symmetric objects. Overall, our approach demonstrates advantages, achieving an increase of approximately 0.3 percentage points. While this percentage increase may seem modest, our proposed method offers substantial flexibility, making it applicable to other approaches. Additionally, our method exhibits good real-time performance. By replacing traditional models with implicit representations and using a constant number of data instead of large feature maps, our approach reduces both data requirements and computation time.

#### 5.3.2. Evaluation on the Occlusion Dataset

**Figure 5:**
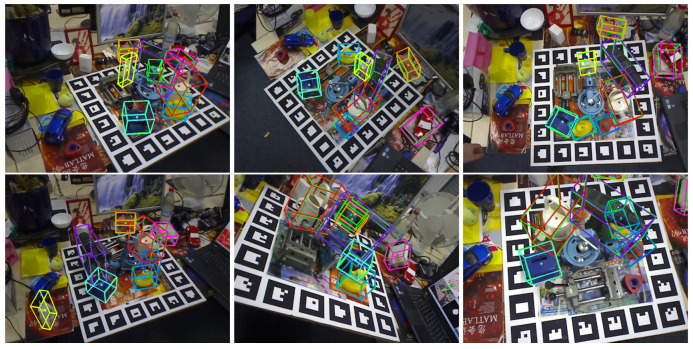
This is multi-object pose estimation on the occlusion dataset. Different objects represent different objects. The two color boxes of each object represent the real box and the estimated box respectively.

In our experiments on occlusion datasets, we demonstrate the effectiveness of our method using multi-object detection, as shown in Figure 5. Our approach performs well in multi-object recognition. Since we employ a testing method consistent with the one proposed for EfficientPose [37], we compare its results with those of our method under different parameters. As shown in Table 2, our method exhibits more pronounced effects on occlusion. For multi-object pose estimation using occlusion datasets, we observe that our ϕ is larger, indicating better model fitting capability and consequently better performance. Comparing our experiments with other methods, we find that our method outperforms others on many datasets. Overall, our method achieves an increase of 1.17 points in average performance. We believe that our method learns different scale features and uncertainty based on implicit representation, enabling better fitting of occluded object poses and thus achieving better results.

### 5.4. Ablation Experiments

In this chapter, we will discuss the effectiveness of the proposed method and compare the experiments conducted after isolating each method. We mainly address two questions: 1. Whether the proposed implicit expression module can improve the effectiveness of pose estimation. 2. Whether the proposed learnable DP-PnP is effective.

#### 5.4.1. Validating the Effectiveness of the Implicit Expression Module

We replaced the implicit expression module with the traditional upsampling operation and compared the pose estimation performance and speed of the two. As shown in the Table 3, with the same number of bidirectional fusion modules and the same backbone network, our method uses the implicit expression module to achieve a more obvious improvement in occlusion data. We mainly compare our method with EfficientPose, modify its corresponding structure to our improved structure, compare traditional upsampling with implicit expression, and compare traditional RT head with our DP-PnP. Compared with the traditional bilinear upsampling method, our method improves the accuracy by 1.14 points when ϕ=0, making substantial progress. At the same time, our method is faster, which means that our method provides a more stable occlusion estimation effect and achieves a faster estimation speed.

#### 5.4.2. To Validate the Effectiveness of DP-PnP

Unlike other learnable PnP methods, our input features are not directly upsampled features but rather features estimated by an implicit module. The uncertainty of the implicit module adds more learnability to the learning process of PnP. By using the learned pose estimation from the learnable PnP to constrain the variables of the implicit module, we achieve better learnability. Through experiments comparing with traditional two-stage methods using traditional PnP for pose estimation, it is evident that our PnP achieves results comparable to or not inferior to traditional PnP. Although our method does not show significant improvement in performance, our single-stage algorithm demonstrates a significant improvement in runtime, indicating the real-time capability and accuracy of our method.

#### 5.4.3. Real-Time Performance Analysis

**Figure 6:**
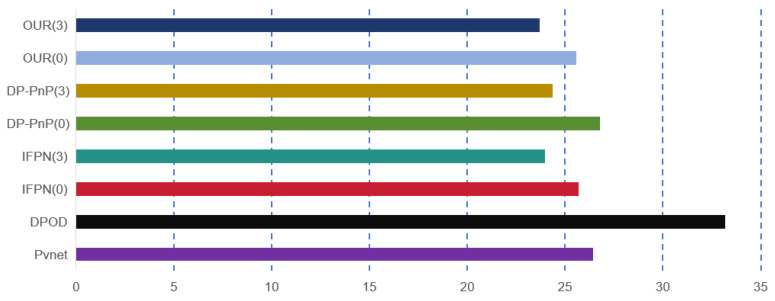
Using the 2080 Ti for training, real-time FPS values for single-object pose estimation were obtained from different network architectures, where the values in parentheses represent the ϕ values of 0 and 3. Additionally, IFPN and DP-PnP denote the inclusion of these modules.

**Figure 7:**
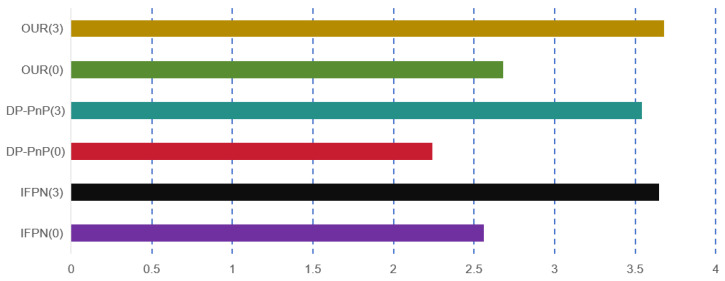
Training single-object pose estimation networks using the 2080 Ti requires time measured in days, where the values in parentheses represent ϕ values of 0 and 3. Additionally, IFPN and DP-PnP denote the inclusion of these modules.

In Figure 6 and Figure 7, we compare our method with several conventional approaches using the 2080 Ti. Considering the impact of experimental conditions, we limited the number of methods compared, focusing on real-time single-object recognition. We found that our method achieves higher accuracy and maintains real-time performance comparable to other methods. This highlights the advantage of our end-to-end approach.

## 6. Conclusions

In our work, we introduced a novel network for estimating the 6D pose of objects. Our main idea involves incorporating implicit representation into our approach, introducing an implicit module that adds the uncertainty of implicit representation to pose estimation. Furthermore, we proposed a new learnable DP-PnP to replace traditional PnP based on this foundation. Our method offers a fast, accurate, and robust 6D pose estimation approach. Additionally, many modules in our proposed method can be applied to other 2D object detection networks and 6D pose estimation networks as portable components or new network design concepts, such as implicit estimation modules and DP-PnP. In future work, we also hope to extend our method to fields such as autonomous driving and robotic grasping.

## Figures and Tables

**Table 1:** The table displays the ADD(s) metrics for individual object pose estimation using different methods for various objects.

Method	YOLO6D	PVNet	DPOP	EfficientPose	RNNPose	Our
Ape	21.62	43.62	53.28	87.71	88.19	87.90
Benchvise	81.80	99.90	95.34	99.71	100	99.94
Cam	36.57	86.86	90.36	97.94	98.04	98.25
Can	68.80	95.47	94.10	98.52	99.31	98.64
Cat	41.82	79.34	60.38	98.00	96.41	98.66
Driller	63.51	96.43	97.72	99.90	99.70	100
Duck	27.23	52.58	66.01	90.99	89.30	91.20
Eggbox *	69.58	99.15	99.72	100	99.53	100
Glue *	80.02	95.66	93.83	100	99.71	100
Holepuncher	42.63	81.92	99.69	100	99.65	97.63
Lamp	71.11	99.33	88.11	100	99.81	99.34
Phone	47.74	92.41	74.24	97.98	98.39	98.23
Averge	55.95	86.27	82.98	97.35	97.37	97.65

Symmetric objects are marked with *.

**Table 2:** The table displays the ADD(s) metrics for multi-target object pose estimation.

Method	Effi (ϕ=0)	Effi (ϕ=3)	Our (ϕ=0)	Our (ϕ=3)
Ape	56.57	59.39	57.65	60.36
Can	91.12	93.27	92.35	93.68
Cat	68.58	79.78	70.56	80.65
Driller	95.64	97.77	95.78	98.35
Duck	65.31	72.21	68.25	73.35
Eggbox *	93.46	96.18	93.45	96.38
Glue *	85.15	90.80	85.96	90.75
Holepuncher	76.53	81.95	77.69	83.12
Averge	79.04	83.98	80.21	84.58

Symmetric objects are marked with *.

**Table 3:** The performance of the ADD(s) metric without the implicit module and DP-PnP.

Method	DP-PnP ϕ=0	DP-PnP ϕ=3	IFPN ϕ=0	IFPN ϕ=3	Our ϕ=0	Our ϕ=3
Ape	57.86	59.63	57.68	59.56	57.65	60.36
Can	91.34	93.28	91.65	92.65	92.35	93.68
Cat	69.37	79.96	68.95	79.65	70.56	80.65
Driller	96.57	97.99	95.96	97.47	95.78	98.35
Duck	63.56	72.69	63.68	72.64	68.25	73.35
Eggbox *	93.18	96.23	93.02	97.15	93.45	96.38
Glue *	85.65	90.70	85.25	90.35	85.96	90.75
Holepuncher	76.96	82.35	76.35	82.65	77.69	83.12
Averge	79.31	84.10	79.07	84.02	80.21	84.58

Symmetric objects are marked with *.

## Data Availability

Data are contained within the article.
